# An Unusual Radiologic Image of Extensive Tumor Mass Infiltrating Hepatic Hilum without Signs of Cholestasis—A Case Report and a Literature Review of Non-Cancerous Lesions Mimicking Intrahepatic Cholangiocarcinoma

**DOI:** 10.3390/curroncol31080336

**Published:** 2024-08-04

**Authors:** Jakub Ciesielka, Krzysztof Jakimów, Ida Cedrych, Anna Kwaśniewska, Jacek Pająk, Jerzy Chudek

**Affiliations:** 1 Student’s Scientific Association, Department of Internal Medicine and Oncological Chemotherapy, Faculty of Medical Sciences in Katowice, Medical University of Silesia, 40-027 Katowice, Poland; krzysztof.jakimow@gmail.com; 2 Department of Internal Medicine and Oncological Chemotherapy, Faculty of Medical Sciences in Katowice, Medical University of Silesia, 40-027 Katowice, Poland; ida.cedrych@gmail.com; 3 Department of Radiology, The Mielecki Hospital, Medical University of Silesia in Katowice, 40-027 Katowice, Poland; akwasniewska@onet.pl; 4 Department of Pathomorphology, Faculty of Medical Sciences in Katowice, Medical University of Silesia in Katowice, 40-027 Katowice, Poland; jacek.pajak@sum.edu.pl

**Keywords:** cholangiocarcinoma, bile duct neoplasms, tuberculosis, hepatic, cholangitis, sclerosing

## Abstract

Background: Mass-forming intrahepatic cholangiocarcinoma (mICC) is the most frequent type of ICC. In contrast-enhanced computed tomography, mICC is visualized as a hypodense lesion with distal dilatation of intrahepatic bile ducts. The presented case illustrates the unusual manifestation of mICC in a 71-year-old male patient, where despite the extensive tumor mass and the hilar infiltration, the dilatation of intrahepatic bile ducts and cholestasis were not noted. Methods: A literature review on PubMed was performed. Primarily, 547 records were identified, and the titles and abstracts were systematically searched. Regarding the inclusion and exclusion criteria, 31 papers describing the non-cancerous liver lesions mimicking ICC were included in the further analysis. Results: In 41.9% of the analyzed non-cancerous lesions, the obstruction of the bile ducts was not noted, similar to our patient. A significant cholestasis has been found in 30.03% of analyzed patients. The invasion of the liver hilum was noted in one-third of the patients. Conclusions: Atypical radiological features in lesions suspected of ICC, such as the absence of intrahepatic bile-duct dilation, are common in benign lesions. In the case of radiologically atypical lesions suspected of ICC, the diagnostic imaging needs to be correlated with clinical data, and the diagnosis should be confirmed with a pathological examination.

## 1. Introduction

Intrahepatic cholangiocarcinoma (ICC) accounts for up to 30% of all liver malignancies and is the second most frequent primary liver tumor with an increasing prevalence worldwide [1,2]. According to the growth pattern and imaging findings, mass-forming ICC (mICC) is the most frequent subtype of ICC [2,3]. The mainstay diagnostic imaging remains contrast-enhanced computed tomography (CT), with a high sensitivity and specificity exceeding 90% in the differential diagnosis of ICC [4]. In CT imaging, mICC is visualized as a well-defined, hypovascular lesion with rim-like enhancement in the arterial phase and staining on the interior in the delayed phase [2]. The pathognomonic feature of extensive ICC, infiltrating the liver hilum, is the dilation of the intrahepatic bile ducts distal to the tumor mass, clinically manifested by cholestasis [5].

In this study, we present an unusual radiologic image of mICC in which, despite the large tumor size and extensive infiltration of the liver hilum, no intrahepatic biliary ductal dilatation, as well as cholestasis, has developed. Given the diagnostic and treatment difficulties of liver lesions resembling ICC, we also conducted a literature review discussing the radiological features of non-cancerous lesions mimicking ICC.

## 2. Case Report

**Figure 1:**
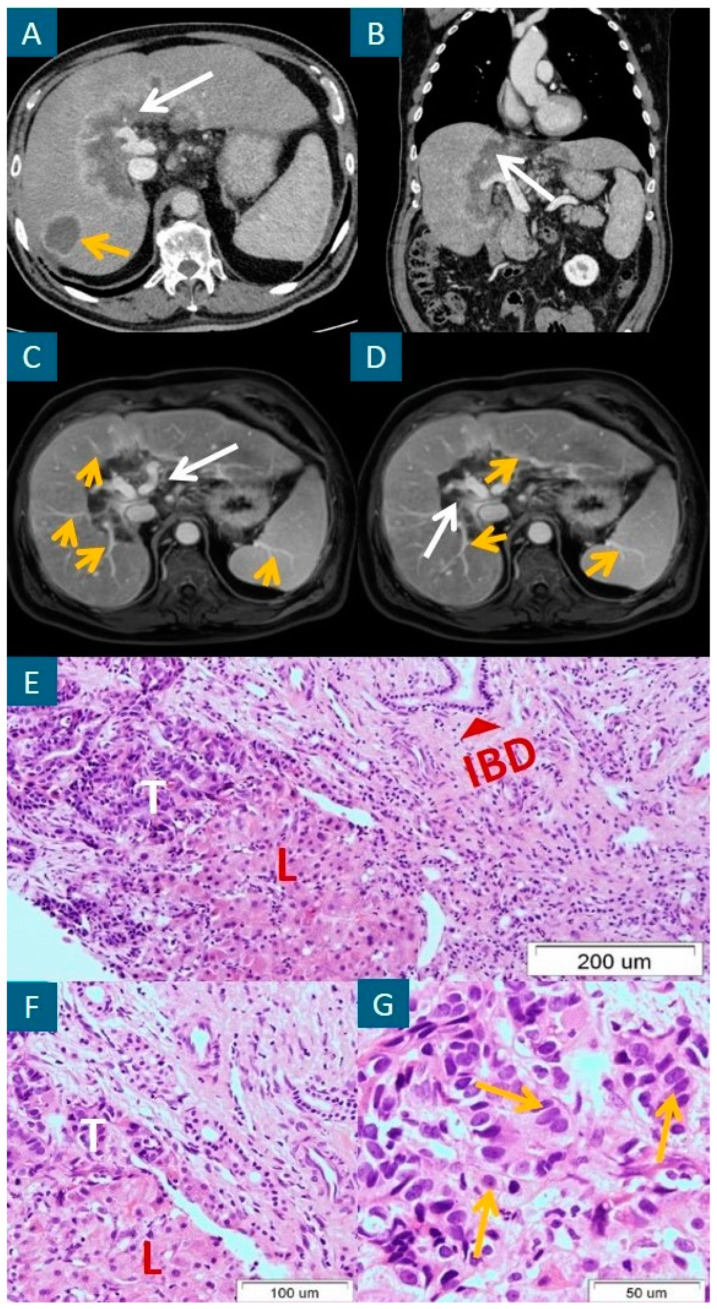
A hypodense extensive lesion located in the hepatic hilum, measuring 123 × 52 mm in size, with a ring-shaped arterial enhancement (white arrow), and a satellite lesion located in the 7th liver segment, measuring up to 45 mm in size (yellow arrow), in the axial (**A**) and sagittal (**B**) sections of the arterial phase of contrast-enhanced computed tomography (CT). The same extensive tumor lesion located in the hepatic hilum (white arrow) without dilation of intrahepatic bile ducts distal to the tumor mass (yellow arrows) in two axial sections of magnetic resonance cholangiopancreatography and T1-weighted fast field echo (T1-FFE) imaging (**C**,**D**). Adenocarcinoma tissue with a normal bile duct on the right upper side of (**E**) (red arrow) and mICC cells (yellow arrows in (**G**)). H&E staining. Original magnification: 100× (**E**), 200× (**F**), and 400× (**G**). T = tumor; L = normal liver tissue; IBD = intrahepatic bile duct.

A 71-year-old male patient without significant comorbidities was admitted for further evaluation of an accidentally found liver lesion in an ultrasound examination. The laboratory work-up revealed a slightly elevated concentration of C-reactive protein (10.4 mg/L; reference range, <3.1 mg/L), ALP (324 IU/L; reference range, <270 U/L), increased values of GGTP (1353 IU/L; reference range, <40 IU/L), and cancer antigen 19-9 (182–215 U/mL; reference range, <37 U/mL). The carcinoembryonic (CEA), and alpha-fetoprotein (AFP) levels were within the reference ranges. Similarly, the total bilirubin concentration remained within a normal range (18.6 µmol/L; reference range, 5.1–20.5 µmol/L). An abdominal CT scan revealed an irregular hypodense lesion located in the hepatic hilum measuring 123 × 52 mm in size with a ring-shaped arterial enhancement and a hypodense satellite lesion measuring up to 45 mm located in the VII liver segment (Figure 1A,B) and numerous enlarged celiac lymph nodes.

The presumptive diagnosis included mICC. However, despite extensive infiltration of the hepatic hilum, there was no obstruction of the intrahepatic bile ducts, as well as their distal dilatation, constituting a pathognomonic feature of infiltrative lesions localized in the hepatic hilum. Regarding diagnostic uncertainties and an asymptomatic course of the disease, it was important to consider extremely rare, atypical hilar lesions that may mimic mass-forming ICC, including primary hepatic tuberculosis and IG4-related sclerosis cholangitis.

In the presented case, the tumor-tissue examination taken from the liver hilum by core needle biopsy provided the diagnosis of small-duct-type mICC G2 (Figure 1E–G), positive for CK19, and CK7, p53 and negative for CK20 and p63. The Ki-67 value was 20%. Notably, the mICC cells did not invade the intrahepatic bile ducts, spreading between hepatocytes and expanding through the sinusoid spaces. Considering the good performance status, the palliative chemotherapy with cisplatin and gemcitabine regimen was administered to the patient with a partial tumor response in follow-up CT scans. A magnetic resonance cholangiopancreatography performed 12 months after the first presentation did not visualize an intrahepatic bile-duct dilatation (Figure 1C,D) corresponding to normal bile drainage and the lack of significant cholestasis. After 22 months of follow-up, the patient continues second-line chemotherapy with capecitabine in monotherapy without any signs of disease progression and symptoms of cholestasis.

## 3. Materials and Methods

### 3.1. Search Strategy

**Figure 2:**
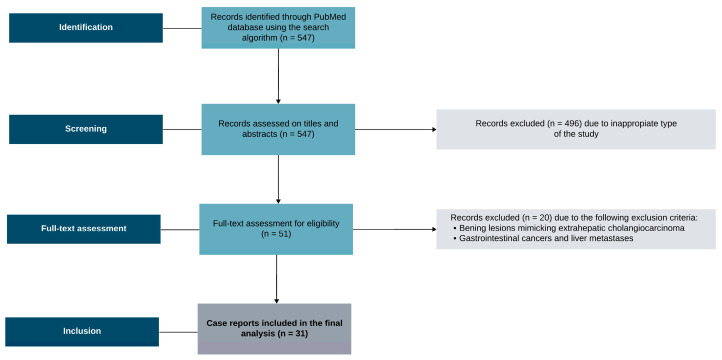
The flowchart for the study selection.

For this review, a search on PubMed was conducted on May 2024, according to the presented search algorithm: [(ICC) OR (cholangiocarcinoma)] AND [(mimicking) OR (resembling)] AND [(lesion) OR (tumor) OR (neoplasm)]. Initially, 547 records were identified and systematically searched on titles and abstracts. Reviews, systematic reviews, meta-analyses, and letters to the editor were excluded. In total, 51 full-text case reports describing the lesions mimicking cholangiocarcinoma were full-text analyzed. Exclusion criteria involved any lesion mimicking extrahepatic cholangiocarcinoma assessed by diagnostic imaging and histologically proven digestive tract cancers, including combined hepatocellular cholangiocarcinoma, hepatic epithelioid hemangioendothelioma, and cancer metastases to the liver. Lymphoproliferative diseases involving the liver parenchyma were enclosed in the analysis. Finally, 31 records were further analyzed. Figure 2 presents the flowchart for the study selection.

### 3.2. Data Acquisition

Each paper was evaluated regarding the patient’s sex, age, tumor localization, and maximal tumor diameter. Radiological features of the lesions were assessed involving CT, magnetic resonance imaging, endoscopic retrograde cholangiopancreatography, and 18F-fluorodeoxyglucose positron emission tomography–CT (18F-FDG PET-CT). The assessed radiological features included bile-duct dilatation distal to the tumor; hilar and vascular invasion; density in plain CT; peripheral rim enhancement; tumor thrombus; capsular retraction, as well as the presence of the satellite lesions; and enlarged lymph nodes. The appropriate histologically proven diagnosis was ordered for each record. The clinical entities were categorized and assigned into seven consecutive categories regarding ‘autoimmune diseases’, ‘infectious diseases’, ‘inflammatory diseases’, ‘hematological diseases’, “benign liver neoplasms’, ‘liver injuries’, and ‘other diseases’. The discrepancies were solved by the consensus.

### 3.3. Statistical Analysis

All statistical analyses were performed using the Jamovi (Version 2.3, 2022) computer software. To assess the normal distribution, Shapiro–Wilk’s normality test was used. Continuous variables were expressed as means (±SD) or medians (±IQR) when appropriate. Categorical data were expressed as numbers (percentages). Analysis of homogeneity of variance was performed using Levene’s test. The data with normal distribution were compared using a *t*-test. Analyzed data that were not normally distributed were compared using the U Mann–Whitney and ANOVA Kruskal–Wallis tests. Statistical significance was stated as *p* < 0.05.

## 4. Results

Out of 31 analyzed patients, females accounted for 51.6% (16/31) and males for 48.4% (15/31) of all analyzed individuals. The age values ranged between 22 and 82 years, with a mean value of 58.6 (SD ± 14.5) years. Females were older compared to males, 62.5 (SD ± 11.5) vs. 54.7 (SD ± 16.5) years, respectively. However, this difference was statistically not significant (*p* = 0.15).

The median of the maximal tumor diameter was 42.5 mm (IQR, 21–65). The tumor diameters in males were higher, compared to females (50 (IQR, 33.8–73) vs. 30 (IQR, 20–45) mm, respectively); however, this observation was without statistical significance (*p* = 0.15). Analyzing the tumor location, we noted that 41.9% (13/31) of all lesions were located in the left liver lobe, 35.5% (11/31) in the right lobe, and 9.7% (3/31) in the caudate lobe. Cholestasis occurred in 30.3% (10/31) of the analyzed patients.

Hilar invasions were found in 35.5% of all cases (11/31). In 22.6% (7/31) of all cases, the authors reported radiological signs of vascular invasion. Distal to the tumor, bile-duct dilatation was found in 58.1% (18/31) of all cases. CT imaging was reported in 67.7% (21/31) of all analyzed lesions. Considering the tumor density in plain CT, 95.2% (20/21) of tumors were hypodense. One identified lesion was isodense with the liver parenchyma. Peripheral rim enhancement was present in 41.9% (13/31) of all analyzed lesions. In none of the analyzed cases was tumor thrombus found. The authors revealed liver capsule retraction in a single patient (1/31–3.2%). The satellite lesions identified by the authors were found in 16.1% (5/31) of cases. Radiologically assessed enlargement of lymph nodes was reported in 12.9% (4/31) of all cases. The detailed characteristic of the assessed cohort is presented in Table 1.

Stratified by the disease category, the highest percentage of females was noted in the inflammatory diseases category, reaching 76.7% (11/13). The highest rate of males was found in the small subgroup with liver injuries (3/3–100%). The highest age values regarding all categories were found in patients with inflammatory diseases. The age values stratified by the disease category did not differ (*p* = 0.33) among the compared groups. The highest median values of maximal lesion diameter were found in patients with hematological diseases and liver injury categories: 75 mm (IQR, 72.5–77.5 mm) and 75 mm (IQR, 62.5–87.5 mm), respectively. The highest percentage of cholestasis was found in individuals with liver injuries and occurred in 66.7% (2/3) of all analyzed cases. In total, 80% (4/5) of patients with a lesion infiltrating the liver hilum developed cholestasis.

In all patients suffering from autoimmune and hematological diseases, distal bile-duct dilatation was present. The highest incidence of vascular invasion was identified in patients with hematological diseases. Peripheral rim enhancement was noticed in all patients with autoimmune diseases. None of the patients was diagnosed radiologically with a thrombus in the tumor area. Most satellite lesions were found in patients with infectious diseases (40%, 2/5). The enlargement of lymph nodes was noticed most frequently in patients with benign liver neoplasms and other disease categories. Table 2 presents the detailed characteristics of the evaluated radiological and clinical features stratified by the disease category.

## 5. Discussion

In our patient, the absence of intrahepatic biliary obstruction, despite the extensive tumor mass, and moderately increased serum CA 19-9 level made the initial diagnosis challenging, causing the necessity to consider non-cancer causes. The long-term assessment of the course of the disease without cholestasis allowed us to suspect that the absence of biliary obstruction may have been attributed to the unusual pattern of ICC cell growth. During tumor development, the mICC cells might not invade the intrahepatic bile ducts, spreading between hepatocytes and expanding through the sinusoid spaces. Additionally, as the tumor originated from small bile ducts, it may have contributed to the preservation of adequate biliary drainage by large bile ducts. These features may account for the absence of cholestasis despite the large tumor size and hilar infiltration in our patient. Moreover, although the tumor required differentiation from mICC at the primary diagnosis, the unusual location of the tumor in the liver hilum drew attention, given that the mICC lesions are more often localized peripherally.

Several authors hypothesized that the absence of periductal infiltration constitutes a favorable prognostic factor correlated with increased survival rates [37]. Similarly, in our case, the lack of intrahepatic bile-duct infiltration in pathological examination and radiological imaging might have contributed to an unusually long progression-free survival and no need for stenting during the diagnosis and long follow-up.

According to the literature review, females represented 51.6% of the ICC patients, being consistent with the epidemiological data worldwide [38]. Dilated intrahepatic bile ducts are a radiological feature of malignant infiltration and should strongly draw attention to ICC as a potential diagnosis [39]. However, intrahepatic bile-duct dilatation was found in our systematic review to be in the high percentage of 58.1% of all non-cancerous lesions mimicking ICC. This emphasizes that the differential diagnosis of tumors with ICC morphology, intrahepatic dilatation of bile ducts, should also draw attention to non-cancerous lesions, which should be considered in the diagnostic work-up. Similarly, nearly half of the diagnosed non-cancerous tumors lack the dilatation of intrahepatic bile ducts. In the case of non-dilated bile ducts, despite the large tumor mass, the differential diagnosis should include intrahepatic non-cancerous lesions, like in our patient.

Despite 10% of cholangiocarcinomas (CCAs) being described as ICC, some perihilar CCAs may have intrahepatic involvement. Thus, perihilar CCAs may be incorrectly classified as ICCs in some cases. This may be confusing when estimating the exact percentage of hilar infiltration in ICC [40]. One-third of the evaluated cases presented invasion of the hilum, which was also described in our patient. It should be noted that perihilar invasion can occur in some benign liver lesions mimicking ICC and must be included in the differential diagnosis. 

Despite the significant intrahepatic bile-duct dilatation, indicating biliary infiltration in 58.1% (18/31) of analyzed cases, cholestasis was found only in 30.3% (10/31) of the assessed individuals. It indicates sufficient biliary drainage, especially when the lesion is located peripherally, explaining the lack of significant cholestasis. Nevertheless, cholestasis constitutes a pathognomonic sign of the liver hilar lesions located above the bifurcation of the left and right intrahepatic bile ducts. Cholestasis has been found in 80% (4/5) of all lesions infiltrating the liver hilum. In one study, Liao et al. described a case of a patient with an ICC-resembling lesion located in the liver hilum, measuring 14 mm in size, involving the left hepatic duct, and radiologically manifesting as bilateral bile-duct dilatation. Despite that, the patient did not develop cholestasis. It indicates the preservation of adequate bile outflow despite the radiological signs of large bile duct involvement. It is consistent with our findings pointing to the disproportion between bile-duct dilatation and clinical cholestasis [6]. Similarly, in our case, the patient did not develop cholestasis; however, contrary to the case described above, we did not find radiological features of bile-duct dilatation despite the involvement of large bile ducts, which is considered a radiological sign of a hilar malignancy. Compared to the case described by Liao et al., it is noteworthy that cholestasis has not developed in our patient despite the significantly higher tumor diameter (123 mm vs. 14 mm). Those features indicate the atypical tumor cell spreading without the involvement of intrahepatic bile ducts.

Regarding the vascular invasion, Nakeeb et al. recorded no vascular involvement in patients with ICC, while in those with perihilar lesions, the encasement of either the hepatic artery or portal vein was seen in 19% [41]. This pathology was visualized in nearly one-fourth of the retrieved cases. In this review, we recorded no tumor thrombosis, consistent with the literature. It is established that CCA is less likely to form a thrombus than HCC [40,42]. Capsular retraction is present in 21% of peripheral ICC; thus, it is considered suggestive of ICC [37,42]. This feature was seen in only one of the cases reviewed and was not present in our patient. A long distance from the liver capsule seems to decrease the chance of capsular retraction in case of the hilar location of the tumor.

The occurrence of satellite nodules in all radiologically assessed ICCs ranged from 68.5% to 99.1% [43]. This was found in our case and 16.1% of the retrieved non-cancerous lesions. Therefore, the presence of satellite lesions in the liver parenchyma largely supported the malignant character of the lesion but can also appear in benign lesions mimicking ICC.

Lymph node metastases may occur in up to 90% of radiologically assessed ICCs [43]. Our patient also presented with lymph node involvement. This pathology was seen in 15.2% of the analyzed non-cancerous lesions. The involvement of lymph nodes, similar to satellite lesions, raises a high suspicion of malignancy, requiring a more profound diagnosis and differentiation from benign entities.

In some of the cases included in the analysis, the authors pointed out that a few patients underwent suboptimal surgical treatment of benign lesions highly suspicious on radiological imaging of being ICC. Given the diversity of benign liver lesions suspected to be ICC, in our review, we support the view of these authors that the decision to qualify for surgical treatment of lesions mimicking ICC should be cautious, considering clinical data, symptomatology, and local epidemiological factors, as in the case of liver tuberculosis [8]. In our patient, radical surgical treatment was not possible at patient referral, and the decision to implement appropriate treatment was made based on a pathological examination. Regarding the presented case, we strongly advocate core-needle biopsy for atypical radiologic lesions before deciding on radical surgical management.

A potential limitation may be adherent to the study. The frequency of the radiological features may be underestimated or overestimated due to the small cohort included in our analysis. Despite these limitations, our study encompasses a variety of non-cancerous lesions mimicking ICC, which should be taken into differential diagnosis in the case of atypical radiological features. Future research should elaborate our findings by performing prospective studies based on large cohorts collected by registries to avoid selection bias.

## 6. Conclusions

Atypical radiological features in lesions suspected of ICC, such as the absence of intrahepatic bile-duct dilation, are common in benign lesions and should be considered in the diagnostic process. Satellite lesions and lymph node enlargement in ICC are not typical in non-cancerous lesions imitating ICC and should raise a suspicion of malignancy. In the case of atypical radiological features of lesions suspected of ICC, the radiological image should be correlated with clinical data and symptomatology, and the diagnosis should be completed with a core biopsy.

## Figures and Tables

**Table 1:** The detailed characteristics of the included hepatic lesions stratified by the disease category.

Sex	Age	Tumor Location	Max. Diameter (mm)	Distal Bile-Duct Dilatation	Hilar Invasion	Cholestasis	Vascular Invasion	Density in Plain CT	Peripheral Rim Enhancement	Tumor Thrombus	Capsular Retraction	Satellite Lesions	Enlarged Lymph Nodes	Diagnosis	Ref.
**Autoimmune diseases**															
F	80	Liver hilum	14	+	+	-	-	NA	+	-	-	-	-	IgG4-related sclerosing cholangitis	[6]
M	49	Right lobe	NA	+	+	-	-	Hypodense	+	-	-	-	-	Autoimmune cholangitis	[7]
**Infectious diseases**															
M	71	Left lobe	45	-	-	-	-	Hypodense	+	-	+	-	-	Macronodular tuberculosis	[8]
M	53	Right lobe	74	-	-	-	-	Hypodense	-	-	-	+	+	Liver tuberculosis	[9]
M	63	Liver hilum	78	+	-	+	-	Hypodense	+	-	-	*-*	*-*	*Klebsiella pneumoniae* abscess	[10]
F	45	Right lobe	NA	+	+	+	-	Hypodense	-	-	-	+	-	Atypical mycobacterial infection	[11]
F	47	Left lobe	NA	+	-	+	-	NA	-	-	-	-	-	Fasciola hepatica	[12]
**Inflammatory diseases**															
F	64	Right lobe	20	-	-	-	-	Hypodense	+	-	-	-	-	Hepatic sarcoidosis	[13]
F	51	Left lobe	20	+	+	-	-	Hypodense	+	-	-	-	+	Hepatic sarcoidosis	[14]
M	72	Left lobe	50	+	+	-	+	NA	+	-	-	+	-	Hepatic inflammatory pseudotumor	[15]
F	56	Left lobe	24	+	-	-	-		-	-	-	-	-	Hepatic inflammatory pseudotumor	[16]
F	75	Left lobe	25	-	-	-	+	NA	+	-	-	-	-	Hepatic inflammatory pseudotumor	[17]
M	80	Left lobe	20	-	-	-	-	Hypodense	-	-	-	-	-	Hepatic Inflammatory Pseudotumor	[18]
F	75	Left lobe	45	+	-	+	-	NA	-	-	-	-	-	Xanthogranulomatous choledochitis	[19]
F	82	Left lobe	40	-	-	-	-	Hypodense	+	-	-	-	-	Hepatic granuloma	[20]
F	63	Right lobe	45	-	-	+	-	Hypodense	+	-	-	+	-	Hepatic granuloma	[21]
F	66	Right lobe, left lobe	181	-	-	-	-	Hypodense	-	-	-	-	-	Ovarian Granulosa Cell Tumor	[22]
F	63	Right lobe	35	-	-	-	+	Hypodense	+	-	-	-	-	Granulomatous hepatitis	[23]
F	60	Liver hilum	NA	+	+	+	-	Hypodense	-	-	-	-	-	Follicular cholangitis	[24]
M	36	Left lobe	NA	+	+	-	+	Hypodense	-	-	-	-	-	Hepatic mucormycosis	[25]
**Bening liver neoplasms**															
F	-	Right lobe	17	-	-	-	-	Isodense	-	-	-	-	+	Focal nodular hyperplasia-like lesion	[26]
M	55	Caudate lobe, right lobe, left lobe	NA	-	-	-	+	Hypodense	+	-	-	-	-	Liver hemangioma	[27]
M	69	Left lobe	30	+	-	-	-	Hypodense	+	-	-	-	-	Multicystic biliary hamartoma	[28]
**Hematological diseases**															
F	53	Right lobe	80	+	+	-	-	Hypodense	-	-	-	-	-	Primary follicular lymphoma	[29]
M	51	Right lobe, liver hilum	70	+	+	+	+	Hypodense	-	-	-	-	-	Primary non-Hodkin’s lymphoma	[30]
**Liver injuries**															
38	68	Right lobe	50	-	-	-	-	Hypodense	-	-	-	-	-	Vascular liver injury	[31]
17	32	Caudate lobe	100	-	-	+	+	NA	-	-	-	-	-	Liver infarct	[32]
NA	22	Liver hilum	NA	+	+	+	-	Hypodense	-	-	-	-	-	Mesalazine-induced bile duct inflammation	[33]
**Others**															
F	57	Caudate lobe, left lobe	NA	+	-	-	-	NA	-	-	-	-	-	Focal Caroli disease	[34]
M	58	Left lobe	20	+	+	-	-	NA	-	-	-	-	+	Heterotopic gastric mucosa	[35]
M	42	NA	NA	+	-	+	-	NA	-	-	-	+	-	Peribiliary cysts	[36]

NA—data not available. Others—hereditary or congenital diseases; conditions of unknown causes unclassified to any disease categories.

**Table 2:** The detailed characteristics of the assessed groups stratified by disease category.

Disease Category	Sex (F—Female; M—Male)	Age (Overall Cohorts)	Max. Tumor Diameter (mm)	Cholestasis	Distal Bile-Duct Dilatation	Hilar Invasion	Vascular Invasion	Peripheral rim Enhancement	Tumor Thrombus	Capsular Retraction	Satellite Lesions	Enlarged Lymph Nodes
Autoimmune	F—50% (1/2)	64.5 (IQR, 56.8–72.3)	14	0%	100% (2/2)	100% (2/2)	0%	100% (2/2)	0	0	0	0
	M—50% (1/2)											
Infectious	F—40% (2/5)	55.8 (IQR, 47–63)	74 (IQR, 59.5–76)	60% (3/5)	60% (3/5)	20% (1/5)	0%	40% (2/5)	0	20% (1/5)	40% (2/5)	20% (1/5)
	M—60% (3/5)											
Inflammatory	F—76.7% (11/13)	64 (IQR, 60–75)	35 (IQR, 22–45)	23.1% (3/13)	46.2% (6/13)	30.1% (4/13)	30.1% (4/13)	53.8% (7/13)	0	0	15.4% (2/13)	7.7% (1/13)
	M—23.3% (2/13)											
Benign liver neoplasms	F—33.3% (1/3)	62 (IQR, 58.5–62.5)	23.5 (IQR, 20.3–26.8)	0%	33.3% (1/3)	0%	33.3% (1/3)	66.7% (2/3)	0	0	0	33.3% (1/3)
	M—66.7% (2/3)											
Hematological	F—50% (1/2)	52 (IQR, 51.5–52.5)	75 (IQR, 72.5–77.5)	50% (1/2)	100% (2/2)	100% (2/2)	50% (1/2)	0	0	0	0	0
	M—50% (1/2)											
Injuries	F—0%	32 (IQR, 27–50)	75 (IQR, 62.5–87.5)	66.7% (2/3)	33.3% (1/3)	33.3% (1/3)	33.3% (1/3)	0	0	0	0	0
	M—100% (3/3)											
Others	F—33.3% (1/3)	57 (IQR, 49.5–57.5)	20	33.3% (1/3)	100% (3/3)	33.3% (1/3)	0%	0	0	0	33.3% (1/3)	33.3% (1/3)
	M—66.7% (2/3)											

## Data Availability

Additional patient data can be obtained from the authors upon reasonable request.
